# A Conversation with Eri Saikawa

**DOI:** 10.1021/acscentsci.2c01545

**Published:** 2023-01-10

**Authors:** Carolyn Wilke

In 2018, Eri Saikawa and her then student Sam Peters started sampling soil around Atlanta. Saikawa, an environmental scientist at Emory University, had recently seen research investigating Atlanta residents’ awareness of heavy-metal contamination in their communities, and she became curious whether the city’s urban agriculture initiative—meant to boost local access to fresh vegetables and fruits—might be inadvertently exposing people to lead.

**Figure:**
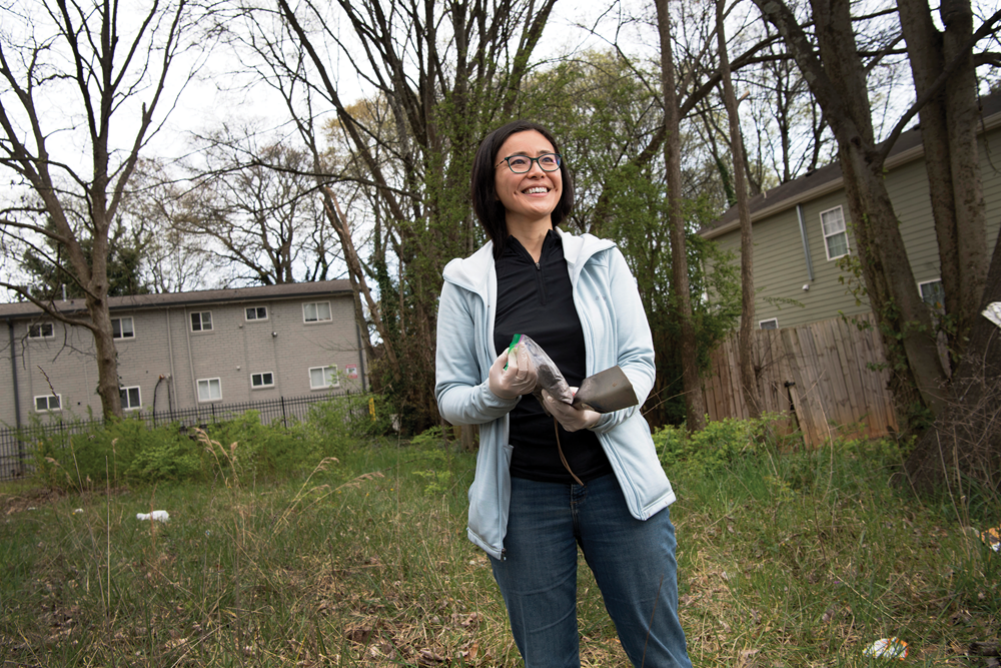
Eri Saikawa collects a soil sample in an Atlanta neighborhood. Credit: Jack Kearse/Emory University

One of their first soil tests suggested that an area of Atlanta known as Westside had unsafe levels of lead, which can slow neurological development, especially in children. So Saikawa’s crew teamed up with the community organization Historic Westside Gardens to sample more locations. Three of 11 residential lots, including ones with gardens, had lead levels above state and federal standards. And in the process of the sampling, Saikawa’s team and Rosario Hernandez, current executive director of Historic Westside Gardens, found mounds of slag—waste material from lead smelting—dumped in the predominantly Black community in the 1950s and ’60s.

With help from US EPA and Westside residents, the sampling effort has grown to over 2,000 lots. Parts of Westside received Superfund status, and in 2022 areas were added to the National Priorities List, which means that the US EPA will clean up the contamination.

Saikawa’s team continues to work in the Westside community. The researchers take blood and soil samples to study the effects of lead and other contaminants and investigate sources of exposure by sampling household dust.

Carolyn Wilke talked with Saikawa about how to involve community residents in public health projects and her mixed feelings about Westside’s Superfund designation. This interview was edited for length and clarity.

## How was the community involved in shaping the goals and the approach of the sampling project?

When we found this high lead level in Westside, Gil Frank, [then] the Historic Westside Gardens executive director, got very interested in working with us to make sure the gardens they have are safe. There was a big mutual interest.

We also found out that a lot of residents have not tested their kids for lead. There was no place where kids can be tested for free or easily in the area. There was a lot of distrust for research and government, and many people said that they didn’t want needles going into their kids. [This distrust stems in part from the US health-care system’s historical and ongoing anti-Black racism, which has resulted in substandard care for Black people in the US—Ed.]

**Figure:**
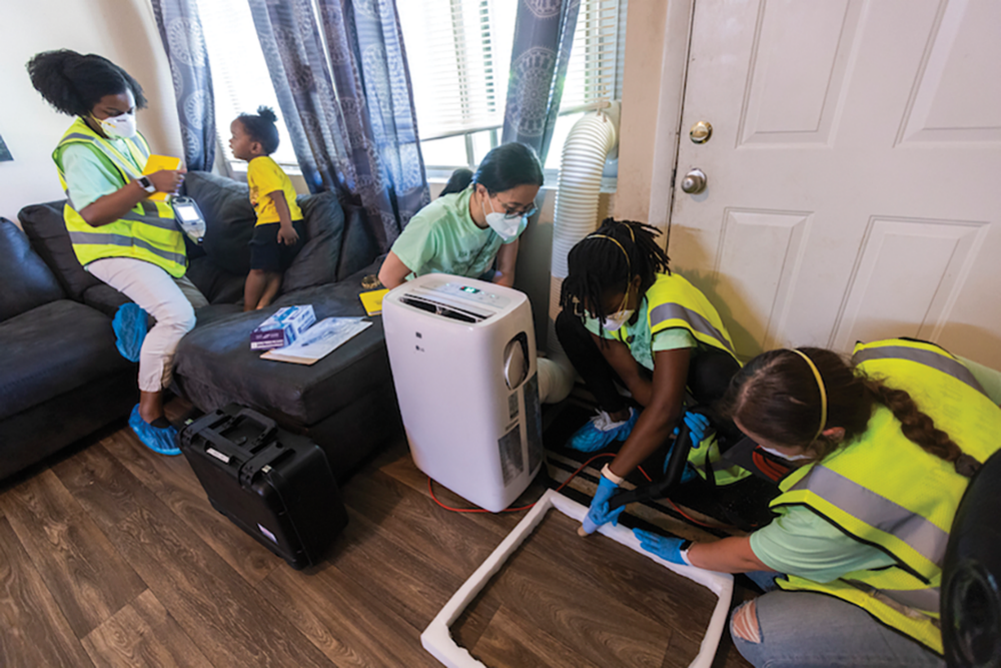
Eri Saikawa and her team collect a dust sample from a home to learn about potential sources of lead exposure. Credit: Kay Hinton/Emory University

Once we learned that they didn’t want [traditional] blood testing, that made us wonder about alternative test methods that would help us understand exposure sources holistically. Now we are testing the soil, paint dust in the household, and the kids’ urine. But even going into the home to vacuum and collect dust samples is very invasive. So I understand that it’s not something that people are necessarily willing to do.

## How did you and your team work to meet community members’ needs?

There was a suggestion of collecting toenails [to measure lead levels]. We created the protocol and then got approved by the Institutional Review Board, but in the end, Rosario was like, “This is not going to work because people find it so gross.”

Instead, we use this equipment called Tasso+. [It attaches to the arm and uses a lancet to take blood near the skin’s surface.] It can take 5 min, but sometimes it takes around a half hour. There is more willingness from parents to take the blood samples that way rather than with needles.

Also, contacting faith groups has been quite successful. Forty-five people agreed to give samples at one event. We also go to different leaders in the community.

## What lessons have you learned about engaging in research with communities that have been marginalized? What advice would you give other researchers?

My training is in atmospheric chemistry, so this was a very unexpected project for me. I wasn’t trained at all to do this kind of work.

I think the university needs to play a bigger role to really integrate with the community. Community engagement doesn’t really fall under the usual grant scheme [for research funding], but if universities have better infrastructure, then it can be used by all researchers, not just for one research project. At Emory, for example, the Hercules Exposome Research Center is trying to create that infrastructure for health research. They put us in touch with Historic Westside Gardens.

And it’s essential to understand how to get opinions from residents in a way that makes sense. Try to get opinions from community leaders when you’re forming the research question.

Getting opinions from the residents themselves is very important too. We rely on the residents to see if [our results are] understandable or not.

## How do you communicate the results to community members?

For soil, it’s pretty simple. There is a 400 ppm EPA safety standard for lead and a 270 ppm Georgia standard. We write residents a letter with their results and use a color scheme. If it’s higher than 400 ppm, we use red. If it’s between 270 and 400, we use orange. If it’s below 270, it’s green.

For blood samples, we want to provide the information that they’re looking for but not raise a false alarm. So we created a visual that has the 50th and 95th percentiles for the US population and the level at which you need to take action. Once you reduce exposure, a decrease in blood lead levels shows up in about a month. [We also include] the range that we saw in the other samples we collected at the same event so they understand where they stood within that population. That came about because of input from residents on what kind of information would be helpful.

## What does it mean to the community and to you that this area of Atlanta received Superfund status?

I’ve been thinking about that quite a bit. When a site becomes a Superfund site, there is usually a huge drop in the property value. Once the site gets cleaned up, property values come back up, but it’s a very long process—it can take 10 years.

This property-value loss could be devastating considering that this is already a low-income area, and residents are overburdened by other environmental and social issues. So I’m sure some people are very upset that this became a Superfund site, and I cannot really say if this was a good thing for the community. I would like to think that by making sure that we don’t have lead contamination in the soil, it will provide a better future.

*Carolyn Wilke is a freelance contributor to**Chemical & Engineering News**, an independent news publication of the American Chemical Society.*

